# Longer commute time is associated with a higher risk of miscarriage: a mixed-effects longitudinal study

**DOI:** 10.1186/s12884-025-08259-8

**Published:** 2025-10-21

**Authors:** Ewa Jarosz, Chen Luo, Anna Matysiak

**Affiliations:** Faculty of Economic Sciences, University of Warsaw, Warsaw, Poland

**Keywords:** Pregnancy, Commuting, Occupational health, Adverse outcomes

## Abstract

**Background:**

Long commuting has been linked to some adverse pregnancy outcomes, but its association with miscarriage, one of the most common pregnancy complications, has not been investigated. This study examined whether the frequency and duration of commuting are associated with miscarriage risk.

**Methods:**

We used waves 1–11 (2008–2019) of the German Family Panel (Pairfam) and sampled women aged 16–45 who had a male partner, worked for pay, and reported a live birth or a miscarriage during the observation period. Cases of recurrent (3 or more) miscarriages were excluded. The final sample consisted of 579 women who reported 458 live births and 121 miscarriages. The association between commuting and miscarriage was examined using mixed effects logistic regression models, stepwise adjusting for work-related confounders.

**Results:**

Commute time longer than 20 min one-way was associated with an increased risk of miscarriage (OR 1.98; CI: 1.00–3.90) compared to commute time under 10 min, in the sample of all commuters. The risk was overall highest for those who commuted daily and for longer than 30 min one-way (OR: 2.28; CI: 1.05–4.98). Commute frequency alone was not associated with an increased risk, but there might be selection into sporadic or no commuting.

**Conclusions:**

A longer commute may represent a modifiable risk factor for miscarriage. Reducing commute time through home-based work, transport-related policies, or flexible scheduling to avoid peak hours could help mitigate this risk, particularly for women with an elevated baseline risk. Future studies should explore potential mechanisms linking commuting to adverse pregnancy outcomes, including stress, environmental exposures, and disruptions to circadian rhythms.

**Supplementary Information:**

The online version contains supplementary material available at 10.1186/s12884-025-08259-8.

## Background

Miscarriage is the most common complication of pregnancy, affecting around one in four pregnant women in their lifetime [1] or around 20% of all pregnancies [2]. These are conservative estimates as many early pregnancy losses are handled at home and not reported [3]. Miscarriage is defined as a loss of pregnancy occurring before the 20th to 28th week of gestation with the cut-off point varying by the research field and world region [4]. Over 50% of miscarriages are caused by foetal chromosomal abnormalities [5, 6], but the rest is unexplained. Miscarriage is associated with adverse physical and mental health outcomes for the mothers and their partners and substantial healthcare and economic costs [4].

As the majority of women of reproductive age are employed, there has been interest in analysing the association between occupational activity and pregnancy complications. Working conditions that are acceptable under other circumstances can increase the risk of adverse pregnancy outcomes, including preterm delivery, low weight for gestational age or miscarriage [7]. While numerous work-related potential risk factors have been examined, to our knowledge, no study has analysed the association between commuting to work and early pregnancy loss. This is a timely topic given the increased availability of remote work following the COVID-19 pandemic, and the frequent discordance between employees’ and employers’ preferences in this regard [8].

### Commuting and women’s health

Research indicates that long commutes negatively affect women’s subjective health, which is also reflected in a higher number of sick leaves taken by female commuters [9]. Although some forms of commuting, such as active commuting (walking or cycling), may offer health benefits due to increased physical activity [10], their prevalence remains low, at around 10%, among adults in developed countries [11].

Studies have established an association between commuting and some adverse pregnancy outcomes, linking longer travel distances with an increased risk of low birth weight and intrauterine growth restriction [9]. For pregnant women, commuting is often a source of significant stress, putting them under physical strain and negatively affecting their wellbeing [10]. It is worth noting that a recent study reported a higher rate of childbearing among first-time mothers who switched to working from home if they would otherwise face a long commute of 40 or more minutes [11]. The causal mechanisms behind this association were not analysed, so it is not known whether a higher rate of childbearing was an outcome of more people deciding to have a child or a result of a higher number of pregnancies ending in live births.

### Possible links between commuting and adverse pregnancy outcomes

There are several possible pathways through which commuting could affect pregnancy outcomes. First, commuting increases maternal stress [10]. Psychological stress disrupts a woman’s menstrual cycle by affecting the hypothalamic-pituitary axis [12]. Several studies have reported that net of other factors, maternal psychological stress is a significant risk factor for miscarriage [13, 14]. Second, travelling to work is a source of exposure to a significant number of air pollutants, including levels of fine particulate matter (PM) that exceed recommended guidelines [15]. Exposure to PM has been associated with an increased risk of spontaneous abortion [16], and high levels of traffic-related air pollution have been associated with higher rates of pregnancy loss [17]. In particular, active commuters (that is, those who walk, run or cycle to work) are exposed to higher doses of pollutants than people using other modes of transportation [18], potentially undermining the health-enhancing effects of active travel. Third, longer commuting decreases individuals’ time available for other activities, such as sleep, rest and leisure, constraining also their time for attending to their health needs, including using antenatal health services [9]. Due to time constraints, longer commuting combined with longer working time has also been linked with making less healthy dietary and lifestyle choices [19]. Finally, commuting may disrupt sleep and lead to waking up before the physiologically optimal time, in particular, if the commute time is long, adding to a woman’s overall physical strain. Work-related strain and disruptions to circadian rhythm have been associated with an increased risk of miscarriage [20, 21].

## Methods

### Data and sample

The aim of this study was to investigate the association between commute time and frequency and the risk of miscarriage. We used the German Family Panel (Pairfam) data [22, 23], waves 1–11, spanning the years between 2008 and 2019. Pairfam uses a population random sample of young adults, starting with three cohorts (aged 15–17, 25–27, and 35–37 years in 2008). The data was collected annually, and we selected women aged between 16 and 45 during the observation period (5,549 women).

The survey included information on live births that happened before each wave and whether a respondent or their partner experienced a miscarriage since the previous wave. There was no clinical assessment of a miscarriage, all data were self-reported. Because the question regarding miscarriage was ambiguous in terms of who experienced the miscarriage (respondent or partner), we retained only respondents who reported having a male partner (4,834 women). Among all women who had a male partner in the preceding wave, there were 1236 live births and 358 miscarriages during the observation period. In this group, the share of miscarriages among all pregnancies was 22%, which is in line with the number reported in other research [24]. There was an expected age gradient in the share of miscarriages to live births (Supplementary materials, Fig. 1S) in the whole sample, with the oldest and youngest women being at a higher risk [4].

To investigate the association between miscarriage and commute, we further restricted the sample to women who worked in the wave preceding live birth or miscarriage. Among working women who miscarried during the entire observation period, over 60% reported having one miscarriage, around 30% reported two miscarriages, and the remaining group reported recurrent (3 or more) miscarriages. Recurrent miscarriages are more likely to be due to underlying health conditions [25], so external factors are a less likely cause. We therefore excluded those cases from the main models but added them back into the sample for robustness checks. Lastly, we excluded women with missing values on the key variables related to commuting. The final analytical sample included 458 live births and 121 miscarriages, with the latter number corresponding to 21% of pregnancies.

### Commuting

Commuting was represented by total commute time one-way (duration) and the number of times a woman commutes to work during the week (frequency). Most women in the sample commuted daily. Over half of the women who commuted to work spent up to 20 min on a one-way commute, and less than 2% of working women declared they always worked from home.

As the association between commute and the risk of miscarriage might be non-linear and dependent on the levels of other variables (e.g. frequency), we collapsed the original continuous duration variable into four categories, roughly corresponding to four quartiles in the distribution in the sample of all working women: commute time of up to 10 min commute one-way (which included zero commute time), 11–20 min; 21–30 min; and over 30 min. We kept the original categories for commute frequency, differentiating between commuting daily, commuting several times per week, and commuting once a week or less often. The distribution of women’s commute time by their commute frequency at time *t* in the final sample of those who gave birth or miscarried at time *t + 1* is given in Table 1.

### Confounders

The first group of confounders was biological factors that increase the probability of miscarriage due to chromosomal anomalies in the foetal tissue karyotype. Maternal age is a main risk factor for miscarriage, in particular, due to an increased risk of chromosomal anomalies among older mothers. However, the general risk of miscarriage is also higher for very young (under 20 years old) women [4, 26]. We used a broader age category [16–23] for the youngest group because of the low incidence of these pregnancies in the data.

Next, the risk is higher for women with a history of previous miscarriages [27]. The modelling strategy accounted for individual-level fixed effects and, in addition to that, we excluded women who reported having multiple (3 or more) miscarriages during the entire observation period. The binary outcome variable did not record consecutive miscarriages if they happened within the same interval between the two waves. We therefore know whether an adverse event happened, but we do not know whether it happened repeatedly for the same woman. This is discussed in the limitations section, though it does not impair the value of our outcome measure, which was designed to capture whether an adverse event occurred or not. Women who miscarried and subsequently became pregnant or gave birth within the same interval between two survey waves were classified as having had a miscarriage [1] because our focus was on the incidence of adverse outcomes, not on whether a miscarriage was ultimately followed by a successful pregnancy.

To account for the time it took a woman to get pregnant, we included the time to pregnancy (TTP). The question regarding TTP was asked to women who were trying to get pregnant. TTP exceeding 12 months is typically classified as subfertility, and it is also an independent risk factor for miscarriage [5]. We differentiate between TTP of under and over 12 months. Many women were not asked about TTP during the interview, for example, because they were not actively trying to get pregnant or they conceived and reported having had a live birth or a miscarriage in the interval between two waves (the waves were, for most of the sample, spaced 10–14 months apart). This category generally implies a short (under 3 months) time to conceive, in contrast to the other two categories, which cover longer trying times (whether under or over 12 months). We refer to these cases as interval conceptions and missing TTP information.

Very low (underweight) or high pre-pregnancy BMI is a risk factor for pregnancy complications, including miscarriage [28, 29]. Information on maternal pre-pregnancy BMI could be derived from the data, but the variable had a high share of missing values. Women with a higher BMI typically also have a longer TTP [26]. We included time to pregnancy in the main models and added pre-pregnancy BMI in the robustness checks, as it substantially lowered our sample size. Lastly, we also controlled for the birth order as there is evidence that nulliparous women are less likely to miscarry than uniparous and multiparous women [30].

Besides the biological factors, a range of work characteristics have been linked with an increased risk of early pregnancy loss. Those include physical or chemical exposures [7, 31] and physical strain [20, 21, 32]. These risk factors often cluster in occupations, such as among workers in the manufacturing or health sector [33]. While we did not have information on all specific risk factors a woman was exposed to, we accounted for occupational differences in the level of risk by including a woman’s occupational class in the models. Original ISCO-08 codes were collapsed into three categories corresponding to three classes: managers and professionals, intermediary, and manual^1^. The cell size for some of the 1-digit ISCO classes was small, and collapsing ISCO codes into three classes prevented model overfitting in our relatively small sample. The overall risk of being exposed to harmful factors or having physically strenuous work was expected to be the highest among manual workers.

Temporal characteristics of work, such as working long hours and working in shifts, in particular night shifts, have been associated with numerous adverse pregnancy outcomes, including miscarriage [34]. We included information on whether a woman worked shifts and whether these were regular shifts or changing time shifts (such as night shifts). Besides its being an independent risk factor for miscarriage, shift work may also confound the effect of commute time since the time of starting and ending one’s job may determine a shorter or longer duration of travel due to it being peak or off-peak. The same may apply to working time, so we differentiated between working part-time, full-time, or more than standard full-time (more than 40 h) per week.

Working time, shift work, and commute time combined may impact a woman’s sleep patterns and leisure behaviours. Leisure-related information, such as the levels of physical activity (PA), was not available in the data, but information on sleep duration was provided. While there is evidence of an association between sleep time and miscarriage [35], sleep duration (unlike waking-up time) might be influenced more by lifestyle preferences or energy levels than working conditions. We therefore did not include sleep duration in the main models but added it in the robustness checks.

Finally, as discussed earlier, commuting might exacerbate maternal stress [10]. The data did not include information on travel-related or work-related stress specifically, but recorded self-reported stress levels during the past four weeks. We included this variable as a risk factor potentially related to employment and travel behaviours. Table 1 shows the distribution of baseline characteristics by commute time.

**Table 1:** Distribution of baseline characteristics at time *t* in the sample of women who miscarried or gave live birth at *t + 1* by commute time

	Total	Commute time in minutes (one way)					
		Up to 10			11–20	21–30	Over 30
	*N* = 579	171			155	108	145
Commute frequency							
Daily	**401**	117			105	74	105
Several times per week	**157**	49			44	33	31
Once a week or less	**21**	5			6	1	9
Age (brackets)							
16–23	**18**	7			4	4	3
24–30	**220**	67			66	31	56
31–36	**260**	75			61	62	62
37–45	**81**	22			24	11	24
TTP							
Under 12 months	**63**	21			12	17	13
Over 12 months	**32**	7			13	6	6
Interval conceptions and not asked	**484**	143			130	85	126
Birth order							
No child born yet	**274**	69			69	52	84
One children	**212**	69			60	38	45
Two children	**93**	33			26	18	16
ISCO							
Managers and professionals	**176**	38			42	38	58
Intermediary positions	**359**	114			105	61	79
Manual and routine	**35**	17			6	7	5
Missing	**9**	2			2	2	3
Work hours per week							
Up to 20	**104**	41			33	17	13
20.5–40	**357**	101			94	68	94
Over 40	**117**	29			27	23	38
Missing	**1**	0			1	0	0
Work schedule							
Only during the day	**410**	116			110	76	108
Fixed shift	**43**	17			11	8	7
Changing shifts	**70**	24			23	9	14
Other or not regular	**56**	14			11	15	16
Stress during past 4 weeks							
Not stressed	**109**	37			34	16	22
Moderately	**92**	33			16	22	21
Significantly stressed	**167**	48			40	35	44
Missing	**211**	53			65	35	58

### Analytical strategy

Using the panel structure of the data, we analysed how commute time and commute frequency during the period preceding the outcome (*t-1*) were associated with the risk of reporting having had a miscarriage in the subsequent wave at time *t*. In our binary outcome variable, 1 represented a miscarriage and 0 represented a live birth.

Associations were tested using mixed effects logistic regression, stepwise adjusting for work-related risk factors. Mixed effects logistic regression models allow for both fixed (within-individuals) and random (between-individuals) effects. We used *melogit* command in Stata 18, which allows for random intercepts and random slopes.

We tested three models. Model 1 used biological determinants of miscarriage risk (age, time to pregnancy, parity), broad occupational class, and the duration and frequency of commuting. Model 2 adjusted for other temporal work characteristics, shift work, total working time, and stress over the past four weeks, which could confound the association between commuting and miscarriage. Model 3 used the specification of Model 2 but narrowed the sample down to women who commuted on a daily basis. Commuting less often than that might mitigate the potential risk posed by commuting. It may also be due to selection – for example, women who experience fertility issues or troubling pregnancy-related symptoms might work from home more often.

## Results

We found the expected associations between the known biological risk factors (maternal age, parity, and time to pregnancy) and the risk of miscarriage among all women. All associations were significant (Supplementary materials, Table 1S).

Model 1 and Model 2 in Table 2 show results for all working women, regardless of their commute frequency. In Model 1, women who spent between 21 and 30 min commuting had a higher risk of miscarriage (OR 1.95; CI: 1.02–3.76), compared to the reference (commute time of up to 10 min). In Model 2, which adjusted for temporal work characteristics and stress, this association remained significant (OR 1.98; CI: 1.00–3.90). We also found an expected positive association between shift work and the risk of miscarriage and a marginally significant lower risk for those who worked reduced hours (< 20). In both Model 1 and Model 2, the effect of commute time that exceeded 30 min one-way was non-significant at 0.05.

**Table 2:** Association between commuting to work and subsequent miscarriage, working women partnered with men, with fewer than 3 reported miscarriages. Results of mixed-effects logistic regression, CI 95%. Coefficients for missing values for categories < 10 cases are not reported

	MODEL 1			MODEL 2		
	OR (SE)	p-value	CI 95%	OR (SE)	p-value	CI 95%
Age (ref. 24–30)						
16–23	5.32 (2.79)	0.001	1.91–14.88	5.66 (3.40)	0.004	1.74–18.39
31–36	0.72 (0.21)	0.246	0.41–1.26	0.73 (0.23)	0.319	0.39–1.36
37–45	3.01 (1.06)	0.002	1.52–5.99	4.23 (1.56)	0.000	2.05–8.71
TTP (ref. <12 months)						
>12 months	2.17 (1.04)	0.105	0.85–5.53	1.69 (0.85)	0.298	0.63–4.55
Interval conceptions and not asked TTP	0.28 (0.09)	0.000	0.15–0.53	0.27 (0.09)	0.000	0.13–0.53
Nr of children (ref. 0)						
One	0.76 (0.21)	0.313	0.44–1.30	0.77 (0.23)	0.385	0.43–1.39
Two and more	1.26 (0.45)	0.518	0.63–2.52	1.17 (0.46)	0.684	0.54–2.53
Occupational class (ref. managers & prof.)						
Intermediary	1.07 (0.28)	0.787	0.65–1.78	1.34 (0.38)	0.296	0.77–2.34
Manual	1.97 (0.91)	0.143	0.79–4.89	2.77 (1.35)	0.037	1.06–7.19
Commute time (ref. <10 min)						
11–20 min	1.47 (0.46)	0.216	0.80–2.72	1.66 (0.54)	0.121	0.88–3.15
21–30 min	1.95 (0.65)	0.045	1.02–3.76	1.98 (0.68)	0.048	1.00–3.90
Over 30 min	1.66 (0.53)	0.115	0.88–3.11	1.71 (0.57)	0.108	0.89–3.29
Commute freq. (ref. daily)						
Several times per week	0.83 (0.22)	0.457	0.48–1.39	0.81 (0.25)	0.494	0.45–1.47
Once a week or less	0.98 (0.62)	0.968	0.28–3.36	0.70 (0.49)	0.604	0.18–2.73
Work hours (ref. < 20)						
20.5–40				0.50 (0.20)	0.079	0.23–1.09
Over 40				0.89 (0.27)	0.713	0.49–1.62
Work schedule (ref. day work)						
Fixed shift				0.95 (0.46)	0.909	0.37–2.45
Changing shifts				3.12 (1.05)	0.001	1.61–6.04
Other, not regular shifts				3.67 (1.35)	0.000	1.78–7.58
Stress (ref. not stressed)						
Moderately stressed				1.04 (0.44)	0.925	0.45–2.39
Significantly stressed				1.85 (0.66)	0.085	0.92–3.71
Missing				1.32 (0.52)	0.485	0.61–2.85
Intercept	0.42 (0.19)	0.056	0.17–1.02	0.21 (0.12)	0.005	0.07–0.63
N obs/N groups (women)	579/477			579/477		

In the sample of women who commuted daily (Table 3, Model 3), the association between time spent commuting and the risk of miscarriage was significant for women whose commute time was longer than 30 min one-way (OR: 2.28; CI: 1.05–4.98). It was close to significant (*p* = 0.089) for those who commute 21–30 min one-way. A lower intercept in the daily commuters group (compared to Model 2 using the same set of independent variables) suggests a lower baseline risk.

**Table 3:** Association between commuting to work and subsequent miscarriage, working women who commute daily. Results of mixed-effects logistic regression, CI 95%. Coefficients for missing values for categories < 10 cases are not reported

	MODEL 3		
	OR (SE)	p-value	CI 95%
Age (ref. 24–30)			
16–23	9.21 (6.51)	0.002	2.30–36.85
31–36	0.6 (0.23)	0.208	0.30–1.30
37–45	5.53 (2.47)	0.000	2.30–13.30
TTP (ref. < 12 months)			
> 12 months	1.64 (1.01)	0.422	0.49–5.50
Interval conceptions and not asked about TTP	0.28 (0.12)	0.004	0.12–0.67
Children born to date (ref. zero)			
One	0.61 (0.23)	0.188	0.30–1.27
Two and more	1.04 (0.50)	0.934	0.40–2.68
Occupational class (ref. managers & prof.)			
Intermediary	1.46 (0.50)	0.271	0.74–2.86
Manual	3.16 (1.90)	0.056	0.97–10.31
Commute time (ref. <10 min)			
11–20 min	1.81 (0.73)	0.414	0.15–2.21
21–30 min	2.07 (0.89)	0.089	0.90–4.80
Over 30 min	2.28 (0.91)	0.038	1.05–4.98
Work hours (ref. under 20)			
20.5–40	0.57 (0.39)	0.414	0.15–2.21
Over 40	0.97 (0.32)	0.923	0.50–1.87
Work schedule (ref. day work)			
Fixed shift	0.89 (0.55)	0.849	0.26–3.01
Changing shifts	3.33 (1.50)	0.008	1.38–8.06
Other, or no regulation of working hours	5.00 (2.32)	0.001	2.01–12.45
Stress (ref. not stressed)			
Moderately stressed	1.32 (0.70)	0.604	0.47–3.71
Significantly stressed	1.97 (0.85)	0.116	0.85–4.57
Missing	1.11 (0.53)	0.824	0.44–2.81
Intercept	0.17 (0.11)	0.008	0.05–0.63
N obs/N groups (women)			401/347

**Fig. 1:**
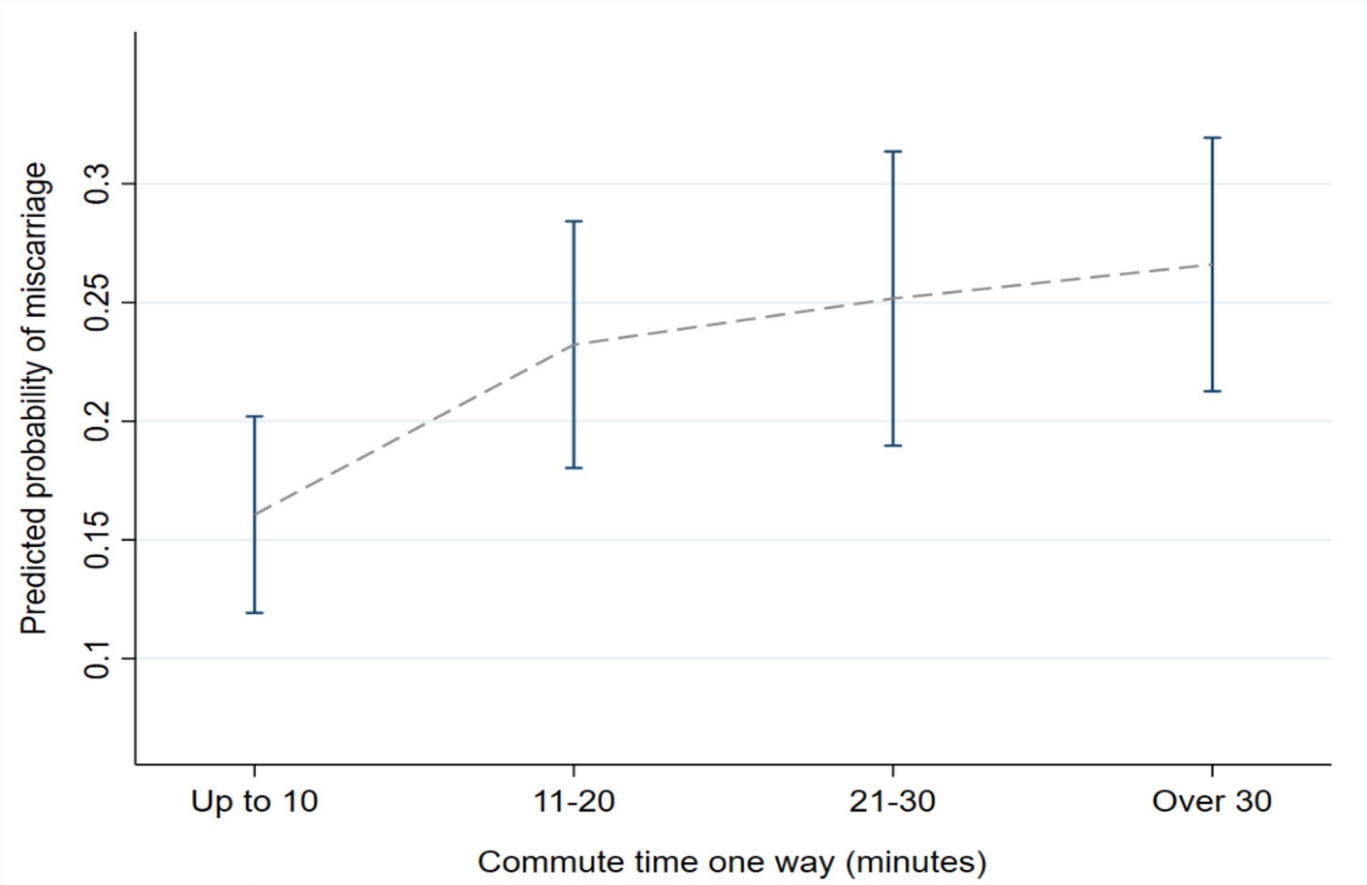
Predictive margins of commute time and miscarriage risk

Figure 1 shows predictive margins for miscarriage risk for daily commuters (Model 3). An 83% confidence interval was used to identify significant differences between the two means with a p-value of 0.05 [36, 37]. The increase in risk was significant for those who commuted 30 or more minutes compared to the reference.

### Robustness checks

We re-run Model 2 and Model 3 (daily commuters only) on a sample of all women, including those who reported multiple (3 or more) miscarriages in the observation period. Longer commute time remained significantly associated with a higher risk of a miscarriage, with this association becoming even more pronounced than in the main models and significant across all levels of commute time greater than 20 min (Table 2S, Supplement). It seems that in the case of multiple miscarriages, the influence of potentially adverse exogenous factors such as lengthy commuting is also worth considering.

Next, we adjusted for the confounders that were not accounted for in the main models, that is, sleep duration and maternal pre-pregnancy BMI. Unlike other variables in the model, BMI was lagged by 2 periods to make sure we capture the pre-pregnancy BMI. Because of the two-year lag and a large number of missing values in the original weight and height variables, the share of missing values in BMI estimates was high, resulting in a lower sample size and greatly inflated standard errors. Due to the low number of cases in different BMI categories, we only ran Model 2 as it offered an overall larger sample. The association between commute time and the risk of miscarriage was significant across all levels of commute, so we conclude it was robust to the different model specifications (Table 3S, Supplement).

## Discussion

This study found a significant association between time spent commuting to work and the risk of miscarriage. Among all working women, the risk was higher for those whose one-way commute exceeded 20 min. Among those who commuted daily, it was higher for women whose commute time was greater than 30 min one way.

There likely is substantial heterogeneity in individual resilience to the effects of commuting and related adjustments in daily travel routines. Some women, such as those in a higher-risk group, might opt out of daily commuting once they become pregnant, especially if the commute is long. The baseline risk of miscarrying was slightly lower for daily commuters compared to all working women, which might point to the healthy worker effect. Those who commuted daily might have been well enough to do so also when pregnant, whereas those who commuted less often might have been doing so because of health or pregnancy-related issues. That would also explain why the risk of miscarriage was significant at a higher ‘dose’ (longer commute time) for women who commuted daily.

These findings contribute to the growing body of literature about potentially adverse health effects of commuting, including its association with pregnancy complications [9]. While we were able to account for the effects of other known occupational risk factors, such as shift work, occupational class (as proxy for some occupational exposures) and total working time, this study has some important limitations. Firstly, all variables, including exposure and outcome, were self-reported. While employed individuals can likely estimate their commute time and frequency with reasonable accuracy, the precision of self-reported time use remains limited. Next, due to a small sample size, we could not examine the effects of some interactions, such as between commute time and commute frequency or between total working time and commute time. The combined load from long working hours and long commuting has been shown to negatively affect an individual’s health behaviours and sleep [38]. Given the increased risk of miscarriage that observed among women with longer commutes, the marginally significant reduction in risk among those working reduced hours (Model 2), and the significantly lower risk among those sleeping eight hours or longer (Model 3 S in the Supplement), the effect of time constraints on maternal health and pregnancy outcomes warrants further investigation.

Related to the above is the fact that there could have been self-selection into certain behaviours (reduced work hours, working from home or commuting less frequently) among some women, perhaps those who were more concerned about pregnancy complications. We were not able to systematically explore the topic due to the limited sample and a low number of measurement points that did not permit observing a woman’s entire fertility history and work-related adjustments she could have been making in relation to childbearing plans. Overall, work-related behavioural modifications remain underexplored in the context of women’s health and fertility.

Due to the survey design, multiple miscarriages occurring in the same interval between the waves were not recorded. While the share of pregnancies ending in miscarriage in our sample was in line with the number reported in other studies, some miscarriages could have been unreported.

Due to data limitations, some key confounders, such as mode of transport (car, public transport, active commute), could not be accounted for. This is an important omission. Different modes of transport are associated with different health risks in terms of the level of exposure to harmful particles, effects on cardiometabolic health or stress levels. Next, environmental exposures are linked to the type of environment in which one travels. We did not have information on the route to work, nor whether travel occurred during peak or off-peak hours, both of which could moderate the effect of commute time by affecting maternal environmental exposures or stress levels.

Lastly, we did not account for maternal health behaviours, such as PA or smoking, as those were either not available in the dataset (PA) or available only for selected waves (smoking). We also did not include any of the father’s characteristics. Though some paternal characteristics, such as his older age [39] and occupational exposures [40], have been linked to the risk of miscarriage, these factors do not differ systematically depending on a woman’s commute time.

Despite its limitations, the study provides new information that may be relevant for the management of pregnancies, particularly those considered high-risk. While awaiting further evidence, practitioners might consider advising women with an elevated risk of miscarriage to adjust their travel routines.

## Conclusions

We found that longer commute time was associated with a higher risk of miscarriage among working women. This may represent a modifiable risk factor, potentially addressed by altering travel behaviours, such as reducing the number of weekly commutes, adjusting commute times, or switching to home-based work. However, the feasibility of such changes may be limited to women in certain occupations. The most effective strategies for mitigating this risk will depend on the underlying mechanisms linking commuting to miscarriage.

Some possible pathways between commuting and miscarriage, such as exposure to pollutants, disruption to circadian rhythm, and stress, have been proposed in this paper, but these are hypothetical and cannot be tested with the available data. Examining the specific mechanisms that may link commuting to miscarriage is important for identifying potential modifications in the mode of transport, time during the day (peak/off-peak) or travel frequency that could help reduce the risk.

## Supplementary Information

Supplementary Material 1.

## Data Availability

The data are available from the pairfam data providers. Syntax files are available from the authors and on the LabFam Zenodo profile: https://www.zenodo.org/communities/labfam/records?q=&l=list&p=1&s=10&sort=newest.

## Footnotes

1 We used the same denotation as in the National Statistics Socio-economic classification (NS-SEC) as it fitted well with our categorization but the empirical basis for clustering into the 3 classes was different.
