# Supplementary material for: Longer commute time is associated with a higher risk of miscarriage: a mixed-effects longitudinal study
Source: BMC Pregnancy Childbirth. 2025 Oct 21;25:1125. doi: 10.1186/s12884-025-08259-8 (PMC12542174; doi:10.1186/s12884-025-08259-8)
Supplement: Supplementary file 1 — Supplementary Material 1. [file 12884_2025_8259_MOESM1_ESM.docx]

**Longer commute time is associated with a higher risk of miscarriage: a mixed-effects longitudinal study**

**Supplementary materials**

Figure 1S. *Number of all reported miscarriages and pregnancies by maternal age, all women with male partners in the wave preceding miscarriage/ live birth.*

Table 1S. *Biological risk factors and the risk of miscarriage: all women. Results of mixed effects logistic regression.*

|  |  | | |
| --- | --- | --- | --- |
|  | OR (SE) | p-value | CI 95% |
| Age (ref. 24-30) |  |  |  |
| 16-23 | 2.25 (0.53) | 0.001 | 1.42 – 3.58 |
| 31-36 | 1.00 (0.16) | 0.976 | 0.73 – 1.37 |
| 37-45 | 1.99 (0.38) | 0.000 | 1.36 – 2.89 |
| TTP (ref. <12 months) |  |  |  |
| >12 months | 2.50 (0.87) | 0.008 | 1.27 – 4.93 |
| Interval conception and TTP not asked | 0.48 (0.11) | 0.001 | 0.30 – 0.75 |
| Nr of children (ref. 0) |  |  |  |
| One | 0.97 (0.15) | 0.826 | 0.71 – 1.32 |
| Two and more | 1.73 (0.32) | 0.003 | 1.20 – 2.48 |
| Intercept | 0.33 (0.08) | 0.000 | 0.21 – 0.54 |
| N obs / N groups | 1,531/ 1,062 | | |

Table 2S. Robustness check 1: *Association between commuting to work and subsequent miscarriage, working women partnered with men, including women with multiple miscarriages (3 or more). Results of mixed-effects logistic regression, CI 95%*

|  | MODEL 2 | | | MODEL 3 (daily commuters) | | |
| --- | --- | --- | --- | --- | --- | --- |
|  | OR (SE) | p-value | CI 95% | OR (SE) | p-value | CI 95% |
| Age (ref. 24-30) |  |  |  |  |  |  |
| 16-23 | 5.63 (3.31) | 0.003 | 1.78 – 17.82 | 9.37 (6.53) | 0.001 | 2.39 – 36.72 |
| 31-36 | 0.72 (0.23) | 0.290 | 0.38 – 1.33 | 0.61 (0.23) | 0.189 | 0.30 – 1.27 |
| 37-45 | 4.46 (1.59) | 0.000 | 2.22 – 8.97 | 5.60 (2.40) | 0.000 | 2.41 – 12.98 |
| TTP (ref. <12 months) |  |  |  |  |  |  |
| >12 months | 2.44 (1.17) | 0.547 | 0.96 – 6.24 | 2.55 (1.48) | 0.105 | 0.82 – 7.93 |
| Interval conception or  TTP not asked | 0.27 (0.10) | 0.000 | 0.14 – 0.54 | 0.29 (0.12) | 0.004 | 0.12 – 0.66 |
| Nr of children (ref. 0) |  |  |  |  |  |  |
| One | 0.84 (0.24) | 0.547 | 0.48 – 1.48 | 0.75 (0.27) | 0.420 | 0.38 – 1.50 |
| Two and more | 1.19 (0.46) | 0.660 | 0.56 – 2.52 | 1.10 (0.52) | 0.841 | 0.43 – 2.80 |
| Occupational class (ref. managers & prof.) |  |  |  |  |  |  |
| Intermediary | 1.44 (0.40) | 0.185 | 0.84 – 2.47 | 1.58 (0.52) | 0.169 | 0.82 – 3.02 |
| Manual | 2.82 (1.37) | 0.033 | 1.08 – 7.31 | 3.30 (1.99) | 0.047 | 1.02 – 10.73 |
| Commute time (ref. <10 mins) |  |  |  |  |  |  |
| 11-20 min | 1.66 (0.54) | 0.120 | 0.88 – 3.12 | 1.82 (0.73) | 0.138 | 0.82 – 4.01 |
| 21-30 min | 2.06 (0.70) | 0.032 | 1.06 – 4.00 | 2.23 (0.93) | 0.053 | 0.99 – 5.04 |
| Over 30 min | 1.77 (0.58) | 0.080 | 0.93 – 3.37 | 2.38 (0.93) | 0.026 | 1.11 – 5.13 |
| Commute freq. (ref. daily) |  |  |  |  |  |  |
| Several times per   week | 0.77 (0.23) | 0.395 | 0.43 – 1.40 | - | - | - |
| Once a week or less | 0.65 (0.45) | 0.538 | 0.17 – 2.56 | - | - | - |
| Work hours (ref. < 20) |  |  |  |  |  |  |
| 20.5-40 | 0.51 (0.20) | 0.093 | 0.24 – 1.12 | 0.54 (0.37) | 0.374 | 0.14 – 2.10 |
| Over 40 | 1.09 (0.32) | 0.757 | 0.62 – 1.92 | 1.25 (0.39) | 0.485 | 0.67 – 2.31 |
| Work schedule (ref. day work) |  |  |  |  |  |  |
| Fixed shift | 0.97 (0.45) | 0.964 | 0.39 – 2.43 | 0.92 (0.54) | 0.892 | 0.29 – 2.93 |
| Changing shifts | 3.16 (1.05) | 0.000 | 1.66 – 6.05 | 3.44 (1.51) | 0.005 | 1.46 – 8.13 |
| Other, not regular shifts | 3.61 (1.32) | 0.000 | 1.77 – 7.38 | 4.89 (2.23) | 0.000 | 2.01 – 11.96 |
| Stress (ref. not stressed) |  |  |  |  |  |  |
| Moderately stressed | 1.07 (0.44) | 0.867 | 0.48 – 2.38 | 1.48 (0.75) | 0.437 | 0.55 – 4.00 |
| Significantly stressed | 1.78 (0.62) | 0.098 | 0.90 – 3.54 | 1.90 (0.80) | 0.129 | 0.83 – 4.36 |
| Missing | 1.26 (0.49) | 0.541 | 0.60 – 2.68 | 1.12 (0.52) | 0.806 | 0.45 – 2.77 |
| Intercept | 0.19 (0.10) | 0.002 | 0.07 – 0.56 | 0.14 (0.09) | 0.003 | 0.04 – 0.51 |
| N obs / N groups (women) | 590/484 | | | 411/354 | | |

Table 3S. Robustness check 2: *Association between commuting to work and subsequent miscarriage, working women partnered with men. Results of mixed-effects logistic regression, CI 95%, adjusting for pre-pregnancy maternal BMI and sleep duration.*

|  | MODEL 2 | | |
| --- | --- | --- | --- |
|  | OR (SE) | p-value | CI 95% |
| Age (ref. 24-30) |  |  |  |
| 16-23 | 4.89 (3.51) | 0.028 | 1.19 – 20.05 |
| 31-36 | 0.72 (0.31) | 0.459 | 0.31 – 1.70 |
| 37-45 |  |  |  |
| TTP (ref. <12 months) |  |  |  |
| >12 months | 2.96 (1.85) | 0.084 | 0.87 – 10.09 |
| Interval conception or TTP not   asked | 0.38 (0.17) | 0.035 | 0.15 – 0.94 |
| Nr of children (ref. 0) |  |  |  |
| One | 0.55 (0.21) | 0.120 | 0.25 – 1.17 |
| Two and more | 0.95 (0.50) | 0.919 | 0.34 – 2.64 |
| Occupational class (ref. managers & prof.) |  |  |  |
| Intermediary | 1.15 (0.42) | 0.690 | 0.60 – 2.34 |
| Manual | 6.33 (4.44) | 0.009 | 1.60 – 25.03 |
| Commute time (ref. <10 mins) |  |  |  |
| 11-20 min | 3.00 (1.45) | 0.022 | 1.17 – 7.72 |
| 21-30 min | 3.53 (1.68) | 0.008 | 1.39 – 8.97 |
| Over 30 min | 2.91 (1.39) | 0.025 | 1.14 – 7.43 |
| Commute freq. (ref. daily) |  |  |  |
| Several times per   week | 0.62 (0.26) | 0.257 | 0.27 – 1.43 |
| Once a week or less | 1.51 (1.99) | 0.757 | 0.11 – 20.17 |
| Work hours (ref. < 20) |  |  |  |
| 20.5-40 | 0.31 (0.17) | 0.035 | 0.10 – 0.92 |
| Over 40 | 0.53 (0.23) | 0.139 | 0.23 – 1.23 |
| Work schedule (ref. day work) |  |  |  |
| Fixed shift | 0.80 (0.56) | 0.751 | 0.20 – 3.18 |
| Changing shifts | 3.29 (1.55) | 0.012 | 1.31 – 8.29 |
| Other, not regular shifts | 3.46 (1.77) | 0.016 | 1.26 – 9.45 |
| Stress (ref. not stressed) |  |  |  |
| Moderately stressed | 0.89 (0.44) | 0.813 | 0.34 – 2.33 |
| Significantly stressed | 1.35 (0.57) | 0.476 | 0.59 – 3.10 |
| Missing | 0.70 (0.39) | 0.525 | 0.24 – 2.07 |
| BMI (ref. normal) |  |  |  |
| Underweight | 18.85 (34.39) | 0.108 | 0.53 – 673.69 |
| Overweight | 19.64 (19.18) | 0.002 | 2.90 – 133.20 |
| Obese | empty |  |  |
| Missing | 3.48 (2.08) | 0.037 | 1.07 – 11.24 |
| Sleep (ref. under 6 hrs) |  |  |  |
| 6 – 8 hrs | 0.66 (0.28) | 0.327 | 0.29 0 1.51 |
| 8 or more hrs | 0.32 (0.15) | 0.014 | 0.13 – 0.94 |
| Intercept | 0.11 (0.11) | 0.031 | 0.02 – 0.82 |
| N obs / N groups (women) | 373/ 320 | | |
